# Iron homeostasis and macrophage polarization in pulmonary fibrosis: mechanisms and therapeutic perspectives

**DOI:** 10.3389/fimmu.2025.1742761

**Published:** 2026-01-15

**Authors:** Minlan Luo, Ali Al-waqeerah, Lili Gao

**Affiliations:** Department of Respiratory Medicine, The First Affiliated Hospital of Dalian Medical University, Dalian, China

**Keywords:** idiopathic pulmonary fibrosis, inflammation, iron metabolism, iron overload, macrophage polarization

## Abstract

Idiopathic pulmonary fibrosis (IPF) is a chronic progressive and fatal interstitial lung disease with limited therapeutic options. Recent evidence highlights dysregulated iron metabolism in macrophages as a critical yet underrecognized driver of disease progression. Excess iron accumulation functions as a signaling cue that promotes macrophage polarization toward the pro-fibrotic M2 phenotype through pathways such as HIF-1α/IL-10/STAT6, contributing to aberrant tissue repair, myofibroblast activation, and excessive extracellular matrix (ECM) deposition. This review synthesizes current findings on the mechanistic interplay between iron homeostasis and macrophage phenotypic switching in IPF and evaluates emerging therapeutic strategies that target iron availability, including iron chelators, ferroportin modulators, and targeted nanocarrier delivery systems. While these approaches show promise, challenges remain regarding specificity, off-target effects, and systemic toxicity. By integrating mechanistic insights with translational advances, this review underscores the therapeutic potential of targeting the macrophage–iron axis and outlines how precision medicine-based interventions may offer novel avenues for effective IPF treatment.

## 1 IPF: a severe clinical challenge

### 1.1 Disease characteristics and substantial burden

IPF is a chronic progressive interstitial lung disease of unknown etiology. Its core pathological mechanism involves persistent alveolar epithelial injury and dysregulated repair processes, accompanied by excessive ECM deposition, ultimately leading to destruction of pulmonary parenchymal structure and progressive decline in gas exchange function (1). Epidemiological studies reveal a pronounced age-related incidence, with incidence rates in individuals over 65 being approximately 11 times higher than in those under 50, indicating that aging is an independent risk factor for the disease (2). Clinically, early symptoms are non-specific, with patients typically presenting insidiously with exertional dyspnea and dry cough, which pose significant challenges for early diagnosis (3–5). According to the latest 2022 American Thoracic Society clinical practice guidelines, diagnosis requires characteristic findings on high-resolution computed tomography (HRCT), supplemented by lung biopsy when necessary (6). The median survival post-diagnosis is typically only 3–5 years. Five-year mortality data indicate a prognosis worse than that of many lung cancers. This grim reality highlights the urgent need for in-depth research into the mechanisms of this disease and the development of targeted therapeutic drugs (7).

### 1.2 Limitations of current therapies and unmet medical needs

Current clinical treatment primarily relies on two classes of targeted drugs: the transforming growth factor-beta(TGF-β) signaling pathway inhibitor, pirfenidone, and the multi-target tyrosine kinase inhibitor, nintedanib (8). Clinical data indicate that pirfenidone can reduce the annual rate of decline in forced vital capacity by approximately 52%. However, concerns arise as over 30% of patients experience adverse reactions such as nausea, anorexia, and photosensitive dermatitis, which significantly impact long-term medication adherence (9). While nintedanib inhibits fibroblast proliferation through multi-targeted action, its use is associated with a 38% increased incidence of diarrhea compared to placebo, along with potential risks of liver function abnormalities (10). It is particularly noteworthy that both drugs can only slow disease progression; they cannot reverse established fibrotic or achieve a cure (11, 12).

For end-stage patients, lung transplantation remains the only treatment option offering the possibility of long-term survival. However, this approach faces multiple challenges, including donor shortage, poor surgical tolerance in elderly patients, and post-operative rejection reactions. Statistics indicate that fewer than 20% of patients meet the criteria for transplantation (13). With the accelerating trend of global population aging, IPF has emerged as a major public health threat, urgently requiring in-depth exploration of its pathogenesis to develop novel therapeutic strategies (14–16). Recent studies indicate that abnormal alveolar epithelial cell repair, immune microenvironment dysregulation, and aberrant intercellular signaling pathways collectively form a complex pathological network in IPF. Investigating these mechanisms may provide new insights for developing more effective targeted therapeutic strategies (17–20).In this network, the pivotal regulatory role of macrophages, which are key innate immune cells, in linking epithelial injury, aberrant immune responses, and the fibrotic process is increasingly recognized (21). Notably, the IPF lung often exhibits pathological features of disrupted iron metabolism, while macrophages are not only primary maintainers of systemic iron homeostasis but also have their functional status significantly influenced by iron metabolism (22). Therefore, an in-depth analysis of the functional plasticity of macrophages and their interaction with iron metabolism offers an important perspective for understanding the pathogenesis of IPF and developing new therapeutic strategies.

## 2 Macrophage functional plasticity: systemic iron recycling and polarization

As key components of the innate immune system, macrophages not only undertake the responsibility of host defense but also play a central role in maintaining organismal homeostasis, with functions far exceeding simple phagocytosis (21). Among these, the systemic iron recycling capacity of macrophages is a key example of their role in maintaining internal environment stability. Tissue-resident macrophages, particularly Kupffer cells in the liver and red pulp macrophages in the spleen, efficiently phagocytose senescent red blood cells and degrade the hemoglobin, thereby recycling and reutilizing iron (23). This process is crucial for the maintenance of systemic iron homeostasis. Additionally, macrophages possess remarkable functional plasticity, enabling them to polarize into phenotypes with different or even opposite functional characteristics based on signals from their microenvironment. This polarization process is referred to as macrophage polarization (24, 25). Among these, classical activation (M1) and alternative activation (M2) are the core models describing their functional states. The M1 phenotype is typically induced by signals such as interferon-gamma (IFN-γ) and lipopolysaccharide (LPS), primarily exerting pro-inflammatory and anti-infective effects; whereas the M2 phenotype is induced by signals such as interleukin-4 (IL-4) and interleukin-13 (IL-13), and is closely associated with anti-inflammatory responses, tissue repair, and fibrogenesis (26, 27). This polarizability is the foundation for macrophages’ participation in various physiological and pathological processes. Understanding these basic concepts is a prerequisite for further discussion of the complex roles of macrophages in specific organs (such as the lungs) and specific diseases (such as IPF).

## 3 Pulmonary macrophage populations

In lung tissue, functionally specialized macrophage populations exist, primarily determined by anatomical location and developmental origin, which collectively maintain pulmonary immune homeostasis (28). Clearly distinguishing these resident populations from the previously described functional polarization states (M1/M2) is essential for understanding the pathogenesis of IPF.

### 3.1 Origin and localization of alveolar macrophages and interstitial macrophages

As key innate immune cells in the lungs, macrophages not only serve as the first line of defense against pathogens and particulate matter but also play a central role in maintaining tissue homeostasis and regulating inflammatory responses. Pulmonary macrophages can be broadly classified into two major types based on anatomical location: alveolar macrophages (AMs) and interstitial macrophages (IMs) (29). AMs originate from erythroid-myeloid progenitor cells during embryogenesis and fetal liver monocytes, reside in the alveolar space, possess self-renewal capacity, and are responsible for clearing foreign substances and apoptotic cells (30, 31). In contrast, IMs, located in the pulmonary interstitium, have distinct origins; some are derived from blood-borne monocytes and perform specialized functions under the guidance of different microenvironmental signals (32). They collectively coordinate the innate immune response in the lungs and contribute to maintaining tissue homeostasis (33).

### 3.2 Phenotypic features and functions under homeostatic conditions

Under homeostatic conditions, AMs and IMs exhibit distinct phenotypic and functional characteristics. AMs are specialized phagocytes distributed in the distal alveolar spaces, responsible for recognizing, phagocytosing, and eliminating invading pathogens, and playing a key role in regulating the initiation and resolution of inflammation, thereby maintaining immune homeostasis at the alveolar surface (31). IMs, due to their high expression of the immunoregulatory cytokine IL-10, possess significant immunosuppressive and regulatory functions (34). Together, these macrophage populations finely regulate pulmonary immune balance (33).

### 3.3 Functional relationship and overlap between macrophage classification and polarization models in IPF

At the pathological level, IPF is characterized by distinct histological changes, including fibroblast foci formation, honeycombing, and disruption of type IV collagen in the basement membrane (35). Its core pathological mechanism involves repeated or persistent alveolar epithelial injury, which activates aberrant repair programs and triggers pathological wound healing characterized by excessive ECM deposition and pulmonary interstitial remodeling (36). In this microenvironment, alveolar epithelial cell injury and its interaction with inflammatory and immune responses are key components of this process (37). It is important to clarify that the classification of macrophages into AMs/IMs (based on anatomical location and origin) and M1/M2 (based on activation state and function) is two different dimensions describing their characteristics, which are not mutually exclusive but are closely related and functionally overlapping (24). In the pathological context of IPF, both AMs located in the alveolar space and IMs residing in the interstitium have the ability to be polarized into M1 or M2 phenotypes based on local microenvironmental signals (26). The disease process of IPF, particularly the stage characterized by fibrosis, creates a microenvironment enriched with cytokines such as TGF-β, IL-4, and IL-13 (38). These signals collectively drive AMs and IMs toward the pro-fibrotic M2 phenotype (39). When AMs and IMs polarize towards the M2 phenotype, they exhibit a common pro-fibrotic role: activated M2-type AMs and M2-type IMs both can secrete large amounts of pro-fibrotic factors, such as TGF-β and platelet-derived growth factor (PDGF), which are core drivers activating pulmonary fibroblasts/myofibroblasts and promoting excessive deposition of ECM (26). This is the core manifestation of the functional overlap between AMs and IMs in the pathological process of IPF. Therefore, numerous studies confirm that macrophages play an indispensable role in the pathogenesis and progression of IPF, functioning not only as immune effector cells but also as key regulators in the fibrotic process.

## 4 Macrophage polarization phenotypes and their dynamic switching in IPF

### 4.1 General regulatory mechanisms and functional characteristics of M1/M2 polarization

Due to their high plasticity, macrophages can differentiate into two functional phenotypes, classically activated (M1) and alternatively activated (M2), based on signals received from their local microenvironment. This polarization process is an important biological mechanism through which macrophages adapt to diverse (24, 25). M1 and M2 macrophages play fundamentally different roles in inflammation and immune regulation (26). The polarization of M1 macrophages is induced by factors such as IFN-γ, LPS, and tumor necrosis factor-alpha (TNF-α) (40). Polarized M1 cells are characterized by overexpression of surface markers such as CD86 and inducible nitric oxide synthase (iNOS), while secreting interleukin-12 (IL-12), TNF-α, reactive oxygen species (ROS), and other pro-inflammatory mediators (41). This polarization process is primarily regulated by signaling pathways such as NF-κB, STAT1, IRF3, and IRF5. In contrast, M2 macrophages are generally considered an anti-inflammatory and pro-repair phenotype, mainly activated by IL-4 and IL-13 (42). Their surface markers include CD163, CD206, and arginase 1 (Arg1), and they effectively suppress excessive inflammatory responses by secreting anti-inflammatory mediators such as IL-10 and TGF-β. Furthermore, chemokines secreted by M2 cells, such as CCL17, CCL18, and CCL22, play key roles in promoting fibroblast activation and ECM deposition, and recruit Th2 cells and regulatory T cells (Tregs) to synergistically regulate immune responses (43, 44). Their polarization primarily depends on the activation of STAT3, STAT6, and peroxisome proliferator-activated receptor delta and gamma (PPARδ/γ) signaling pathways (45–48).

### 4.2 Dynamic switching of polarization phenotypes and their pathological contribution in the course of IPF

In the pathogenesis of IPF, the polarization state of macrophages undergoes dynamic switching along with the disease stages. In the early stage of the disease or during acute exacerbations, M1 macrophages may dominate; their sustained activation exacerbates inflammatory responses and leads to lung tissue damage (49). Additionally, M1 cells extensively degrade ECM components by producing matrix metalloproteinase-9 (MMP-9), directly participating in the pathological destruction of the lung parenchyma (50). As the disease progresses to the chronic fibrotic stage, M2 macrophages gradually become the dominant population (38, 51). At the molecular level, stimuli such as IL-4 induce STAT6 phosphorylation, thereby driving the pulmonary fibrosis process (52). At the cellular level, M2 macrophages interact with epithelial cells, endothelial cells, and resident fibroblasts to collectively promote the formation of a pro-fibrotic microenvironment. These cells further disrupt the balance between ECM synthesis and degradation by secreting matrix metalloproteinases (MMPs) and their tissue inhibitors (TIMPs) (53). Although in the IPF disease state, AMs and IMs often predominantly exhibit an M2 polarization phenotype and dominate the fibrotic process, this does not exclude their potential to polarize towards the M1 phenotype under specific microenvironmental stimuli (such as co-existing acute infection) (54). However, in the pathological stage of IPF dominated by chronic fibrosis, the pro-repair/pro-fibrotic functions mediated by the M2 phenotype are more prominent, thereby causing macrophages as a whole to exhibit significant M2-related characteristics, collectively driving disease progression.

## 5 Dysregulated iron metabolism: a core link in the pathological process of IPF

### 5.1 Fundamentals of iron homeostasis and its general impact on immune cells

Iron is an essential transition metal for biological processes. Due to its variable valence states, it participates in various electron transfer reactions, playing a vital role in maintaining normal physiological functions (55). The maintenance of systemic iron homeostasis relies on a complex regulatory network involving multiple processes such as iron uptake, transport, storage, and efflux. Within this network, transferrin receptor 1 (TfR1) mediates the endocytosis of the transferrin-iron complex, after which divalent metal transporter 1 (DMT1) transports iron ions into the cytoplasm (56). Intracellular iron is stored primarily as ferritin, a spherical structure composed of heavy (FTH1) and light (FTL) chains. Iron efflux depends on the membrane iron exporter SLC40A1 (ferroportin) (57), whose expression is finely regulated by iron regulatory proteins (IRPs) and the hormone hepcidin (58). In immune cells, iron ions serve as cofactors for numerous proteins, participating in critical biological processes such as oxygen carrier synthesis, macromolecule metabolism, and cellular energy production. Therefore, maintaining iron homeostasis is crucial for health, as both iron deficiency and overload can lead to severe pathological conditions (59). Iron availability can influence the metabolic reprogramming of macrophages, thereby regulating their polarization state. Studies show that iron accumulation activates the HIF-1α/IL-10/STAT6 axis, promoting macrophage polarization towards the M2 phenotype and increasing the expression of M2 markers such as ARG1 and CD206 (60, 61). This fundamental regulatory mechanism is the cornerstone for understanding the role of iron in pathological conditions.

### 5.2 Cell-type and microenvironment specificity of iron metabolism regulation in macrophages

Iron metabolism exhibits significant complexity and context-dependency in regulating macrophage polarization. This difference may be related to the metabolic characteristics and microenvironmental localization of different macrophage subsets (62–64). For example, exogenous iron supplements, including ferric ammonium citrate (FAC, 0.25 mM Fe^3+^), ferric citrate (2.5 mg/ml, ~10.2 mM Fe^3+^), ferrous citrate (2.5 mg/ml, ~10.16 mM Fe^2+^), and iron salts (Fe_3_H_2_O_4_, 2.73 mg Fe/ml, ~48.9 mM Fe^2+^), can modulate polarization in different macrophage models: they can upregulate M1 markers (such as iNOS, TNF-α, and IL-1β) in RAW264.7 cells (65, 66), and similar or different effects are observed in bone marrow-derived macrophages (BMDMs) (67) and THP-1 cells (68). However, within the 0–1 mM range, Fe^2+^ delivered as FeSO_4_ significantly inhibits nitric oxide (NO) synthesis in mouse and rat macrophages co-stimulated with LPS and IFN-γ, potentially downregulating M1 macrophage polarization (69). Alterations in iron metabolism during macrophage polarization also vary across different diseases and microenvironments. For instance, in non-alcoholic fatty liver disease (NAFLD), treatment with exogenous iron FAC increases M1 marker expression levels while decreasing M2 marker expression, thereby exacerbating macrophage-mediated inflammatory responses and fibrosis progression (67). Some studies propose that iron overload induces increased M1 polarization of macrophages at different stages. In early liver fibrosis, it promotes increased M2 polarization, whereas in the middle and late stages, it exerts a negative regulatory effect on M2 polarization (70). In the remodeling of acute myeloid leukemia (AML), iron overload can alter the immune landscape of the microenvironment by inducing leukemia-associated macrophages (LAMs) towards M2 polarization (71). Although multiple studies confirm the association between iron metabolism and M2 polarization, others indicate that elevated ferritin regulation during iron overload suppresses STAT6, potentially reducing the induction of M2 polarization in macrophages (65, 72, 73). However, Ali and colleagues demonstrated that in pulmonary interstitial fibrosis, macrophages overexpressing TfR1 exhibit an M2-like phenotype with pro-fibrotic properties, and the number of these macrophages significantly decreased after intervention with the iron chelator deferoxamine (DFO) (62). Ahoro and the GAN team share the same view. Their research shows that iron influences inflammatory processes by regulating immune cell function. Elevated iron concentrations promote macrophage differentiation towards the M2 phenotype and inhibit LPS-induced M1 pro-inflammatory responses (74, 75). Furthermore, alterations in ATF4 expression are significantly associated with M2 macrophage polarization (76). Acute iron deficiency decreases ATF4 expression, suggesting that iron deficiency may disrupt M2 macrophage polarization (77). Studies on mitochondrial reactive oxygen species (mtROS) suggest that iron supplementation may regulate M2 macrophage polarization by modulating the GMFG/mtROS/NF-κB axis (78). Another related study indicates that iron overload suppresses GMFG expression (72), and functional blockade of GMFG downregulates the protein levels of NDUFV2 (Complex I), SDHD (Complex II), and SOD (79). These findings suggest that iron may negatively regulate M2-like macrophage polarization through the GMFG-OXPHOS signaling axis. Therefore, the final impact of iron supplementation or overload on macrophage polarization depends on a complex balance among multiple intracellular signaling pathways (80). This complexity highlights the necessity of interpreting the role of iron metabolism within specific pathological contexts.

### 5.3 Ferroptosis: a specific cell death mode triggered by iron overload in IPF

In IPF, disrupted iron homeostasis not only impairs cellular function but can also directly induce a novel iron-dependent form of cell death known as ferroptosis (61). Iron acts as the central executor of ferroptosis; its redox-active properties catalyze ROS generation through the Fenton reaction, which initiates lipid peroxidation of cellular membranes, ultimately resulting in membrane damage and cell death (81). Cellular iron balance is governed by tightly regulated pathways involving iron import (e.g., TfR1), export (e.g., ferroportin), and storage (e.g., ferritin). Disruption of this balance promotes the accumulation of labile iron, thereby sensitizing cells to ferroptosis (82). A pivotal mechanism controlling intracellular iron levels is ferritinophagy—the selective autophagic degradation of ferritin mediated by nuclear receptor coactivator 4 (NCOA4). This process releases stored iron into the cytosol, increasing cellular susceptibility to ferroptosis (83). Recent evidence indicates that enhanced ferritinophagy in alveolar epithelial cells drives iron overload and ferroptosis in experimental models of pulmonary fibrosis (84). In IPF patients, elevated lung iron levels and altered expression of iron-handling proteins are observed, particularly within fibrotic regions (85). Interventions that reduce iron accumulation or inhibit ferritinophagy—such as treatment with DFO or NCOA4 knockdown—have been shown to attenuate epithelial cell death and collagen deposition in bleomycin-induced fibrosis models, supporting a pathogenic role for iron dysregulation and ferroptosis in IPF (83). Collectively, these findings underscore that aberrant iron metabolism—characterized by excessive iron import, accelerated ferritin degradation, and impaired iron export—serves as a key trigger for ferroptosis in IPF (61).

### 5.4 Core mechanisms of iron homeostasis dysregulation and integrated pathogenic effects in IPF

**Figure 1:**
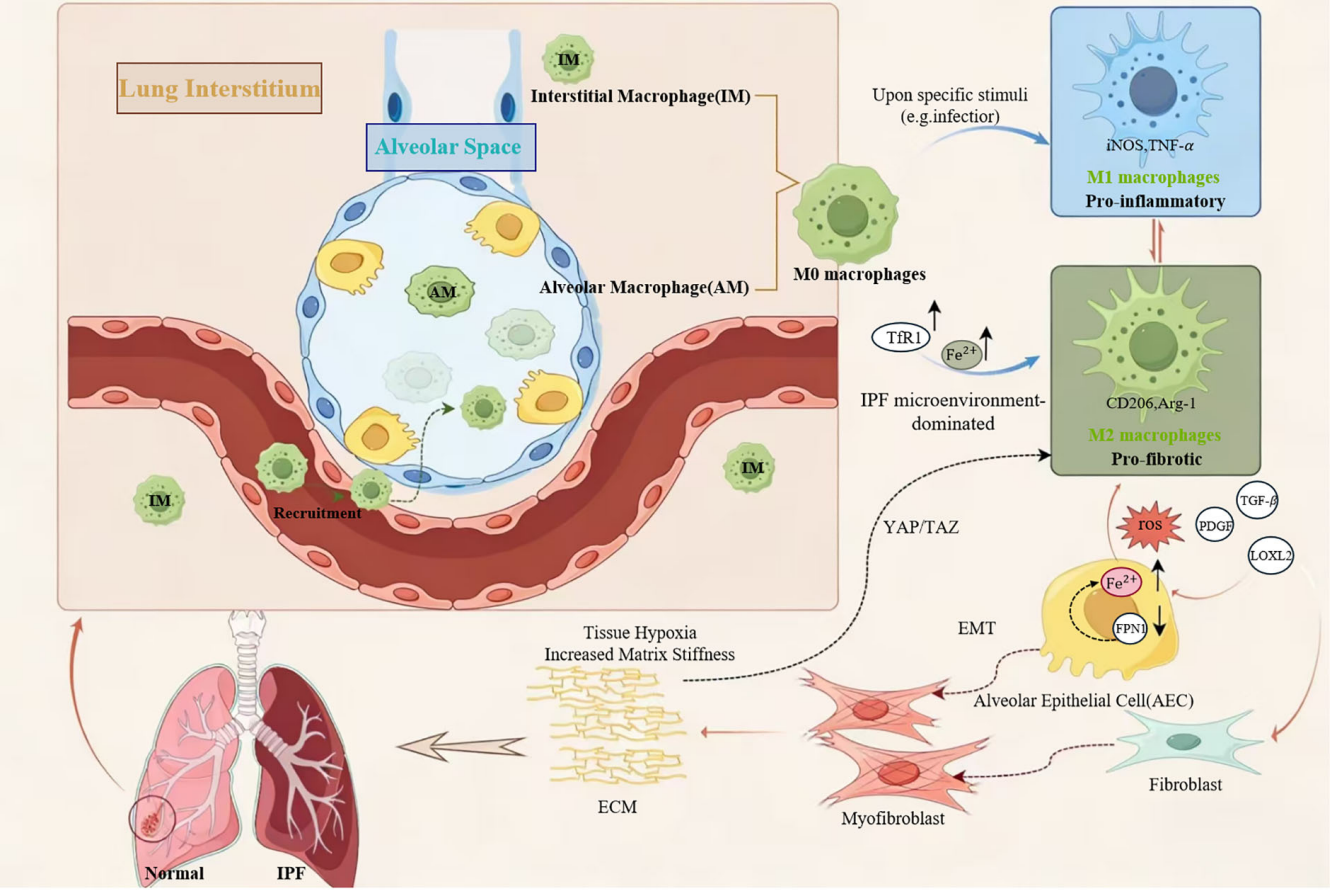
Integrated pathological model of iron dysregulation and macrophage polarization in IPF. This illustration delineates the core events and the self-reinforcing vicious cycle in the pathogenesis of IPF. The process is initiated by iron overload resulting from dysregulated iron homeostasis (e.g., downregulation of the iron exporter ferroportin/FPN1 in alveolar epithelial cells and upregulation of the TfR1 in macrophages). This disruption in iron homeostasis drives the polarization of both AMs and IMs towards a pro-fibrotic M2 phenotype. Activated M2 macrophages secrete copious amounts of pro-fibrotic mediators (e.g., TGF-β, PDGF, LOXL2), which directly activate fibroblasts and promote their differentiation into ECM-producing myofibroblasts. The consequent excessive ECM deposition induces tissue stiffness and hypoxia, which in turn further disrupts iron homeostasis and reinforces M2 macrophage polarization, thereby establishing a self-amplifying cycle that fuels disease progression. Key molecules and cellular processes are annotated.

**Figure 2:**
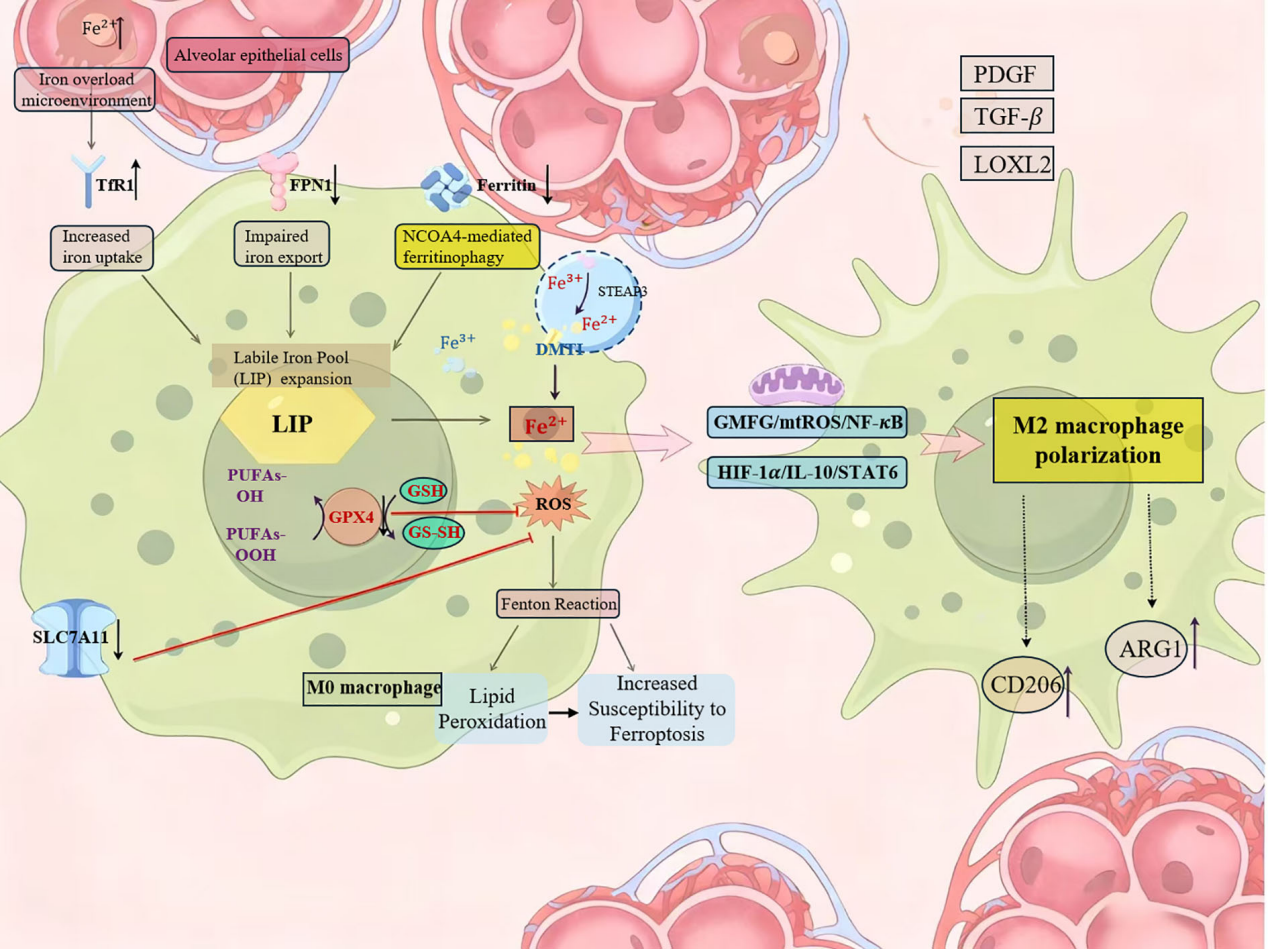
Molecular mechanisms of iron overload-induced ferroptosis and macrophage M2 polarization in IPF. This schematic details the intracellular iron overload in macrophages caused by microenvironmental iron homeostasis imbalance, highlighting its potential role in IPF. The core process involves the expansion of the LIP within the cell. The elevated iron generates lipid ROS via the Fenton reaction.The primary antioxidant defense system, comprising the cystine/glutamate antiporter (system xc-) and glutathione peroxidase 4 (GPX4), prevents ferroptosis by reducing lipid peroxides. Iron overload increases cellular susceptibility to ferroptosis. Furthermore, excess iron and ROS can promote macrophage polarization towards an M2 phenotype via the GMFG/mtROS/NF-κB and HIF-1α/IL-10/STAT6 pathways, respectively, leading to the release of pro-fibrotic factors.

The core pathological cycle driven by iron dysregulation in IPF is summarized in **Figure 1**, which illustrates the integrated vicious cycle of iron overload, macrophage polarization, and fibrotic progression. In IPF, chronic obstructive pulmonary disease (COPD), and acute lung injury (ALI), dysregulated iron metabolism and associated redox imbalance are important factors in disease onset and progression (86–88). During the pathogenesis of IPF, iron homeostasis imbalance manifests as dysregulation across multiple pathways (89). In the pulmonary microenvironment, alveolar epithelial cells and macrophages jointly participate in maintaining iron homeostasis (22). When extracellular iron ion concentrations are abnormally elevated, the internal homeostasis of the lung tissue is disrupted. This manifests as impaired iron sequestration capacity in macrophages and dysregulation of iron uptake mechanisms in alveolar epithelial cells, ultimately leading to excessive intracellular iron accumulation (89). Downregulation of SLC40A1 expression in alveolar epithelial cells impairs iron efflux, while upregulation of TfR1 expression in macrophages enhances iron uptake, resulting in intracellular iron accumulation. Enhanced heme degradation within pulmonary macrophages can cause localized iron overload, while upregulated DMT1 expression further exacerbates iron accumulation in alveolar cells (90, 91). Iron overload in AT2 cells initiates a transcriptional cascade, disrupting iron homeostasis and subsequently affecting macrophage recruitment (92). Iron overload generates ROS via the Fenton reaction, triggering lipid peroxidation and protein denaturation, directly damaging alveolar epithelial and endothelial cells (93, 94). More critically, the disrupted iron homeostasis activates multiple fibrosis-related pathways, including upregulation of TGF-β/Smad signaling and stabilization of hypoxia-inducible factor HIF-1α, collectively promoting fibroblast activation and excessive ECM deposition (93, 95). Polarized M2 macrophages subsequently secrete pro-fibrotic factors such as TGF-β,PDGF, and lysyl oxidase-like 2 (LOXL2), directly driving epithelial-mesenchymal transition (EMT) and fibroblast activation (96). Conversely, features of the pulmonary microenvironment significantly influence macrophage iron metabolism. Hypoxia enhances iron’s effects by stabilizing HIF-1α, while increased matrix stiffness activates the YAP/TAZ pathway via the mechanosensitive ion channel Piezo1, synergistically amplifying the fibrotic response to iron signaling (61, 97) Therefore, iron homeostasis dysregulation in IPF forms a self-reinforcing vicious cycle: it begins with iron overload-induced epithelial cell injury and macrophage iron overload, characterized by impaired iron sequestration in macrophages and dysregulated uptake in alveolar epithelial cells, promoting macrophage polarization towards the M2 phenotype; the pro-fibrotic factors secreted by M2 macrophages then activate fibroblasts, leading to excessive ECM deposition, tissue stiffness, and hypoxia, which in turn further disrupt iron homeostasis, exacerbating the initial iron overload. In summary, iron metabolism dysregulation in IPF, by driving macrophage M2 polarization and direct cellular damage, forms a self-reinforcing vicious cycle with the fibrotic process. At the molecular level, the detailed mechanisms through which iron overload concurrently triggers ferroptotic cell death and promotes M2 macrophage polarization are delineated in **Figure 2**. These findings provide new insights for developing therapeutic strategies targeting iron metabolism.

## 6 Targeting the iron metabolism-macrophage axis: intervention strategies and translational prospects

Various potential therapeutic strategies targeting the iron metabolism-macrophage axis are summarized in **Table 1**, which classifies these strategies and their mechanisms. This chapter will discuss them in detail.

**Table 1:** Summary of potential therapeutic strategies targeting the iron metabolism-macrophage axis in IPF.

Category of intervention	Specific agent / approach	Primary target / proposed mechanism
Reducing Intracellular Iron Levels	Deferoxamine (DFO)	As an iron chelator, reduces intracellular iron levels in macrophages, leading to decreased expression of M2 markers (CD206, ARG1) (98).
Modulating Iron Transport	Hepcidin (agonist)	May restore iron efflux mediated by the iron exporter SLC40A1 (ferroportin), thereby alleviating iron overload (100).
Inhibiting Ferroptosis	Ferrostatin-1 (Fer1)	Inhibits ferroptosis. In models, suppresses the enhancement of pulmonary inflammation and fibrosis induced by the combined effect of the SARS-CoV-2 spike protein and ferric ammonium citrate (FAC) (72).
Activating Antioxidant Pathways	Nrf2 agonist (Dimethyl fumarate, DMF)	Reduces aberrant extracellular matrix (ECM) deposition by activating the Nrf2 signaling pathway. Delivered using ROS-responsive liposomes (DTP@DMF NPs)[l02].
Modulating Heme Metabolism	H0-1 inhibitor	HO-1 is the central enzyme in heme degradation, catalyzing the conversion of heme to carbon monoxide (CO), biliverdin, and free ferrous iron. Its downregulation impairs the M2 polarization capacity of macrophages (106).
Combination Strategies	DFO + Esomeprazole (PPI)	Delays fibrosis progression by intervening in macrophage-fibroblast interactions and modulating iron homeostasis (109).
Advanced Targeted Delivery Systems	Nanoparticles / Inhalable formulations	Offer potential solutions for improving lung tissue targeting while reducing systemic exposure (111, 112).

This table categorizes and summarizes emerging intervention strategies aimed at modulating the iron-macrophage axis to counteract fibrosis in IPF. Strategies are classified based on their primary approach, such as modulating iron transport, inhibiting ferroptosis, regulating iron metabolism-related proteins, and employing advanced targeted delivery systems. For each strategy, the table lists representative agents or approaches along with their postulated primary mechanisms of action. The table provides a concise overview of the current therapeutic exploration landscape in this field.

### 6.1 Direct intervention strategies for iron metabolism and their challenges

Targeted iron metabolism inhibition strategies demonstrate potential therapeutic value in IPF, but their clinical translation requires a deep understanding of the iron homeostasis regulation mechanisms in the pulmonary microenvironment. Existing research indicates that interventions targeting macrophage iron ion concentrations may influence fibrosis progression by modulating immune polarization states. For example, in THP-1 cells, the SARS-CoV-2 spike protein promotes the expression of IL-6 and S100A9. This effect is enhanced by FAC treatment but suppressed by DFO or Ferrostatin-1 (Fer-1) intervention, thereby inhibiting pulmonary inflammation and fibrosis (93). The iron chelator DFO has been shown in preclinical studies to significantly reduce intracellular iron concentrations in macrophages, leading to decreased expression levels of M2 markers CD206 and ARG1 (62, 98). However, systemic iron chelation therapy still faces significant challenges, including the risk of anemia due to nonspecific iron depletion and other systemic side effects, which severely limit its clinical application potential (99).

Another strategy could focus on regulating molecules involved in iron metabolism mechanisms, such as hepcidin, a key regulator of iron metabolism, which may restore iron efflux mediated by the membrane iron transporter SLC40A1, thereby alleviating iron overload (100). Alternatively, ferroptosis inhibitors could be employed to block iron accumulation in pulmonary macrophages, thereby suppressing subsequent lipid peroxidation and TGF-β1 expression, and preventing the activation of pulmonary fibroblasts (101). Improvements in IPF can also be achieved by regulating other factors produced during disrupted iron metabolism, such as the well-known ROS. Current research confirms that ROS-responsive liposomes delivering the Nrf2 agonist dimethyl fumarate (DMF) (DTP@DMF NPs) can inhibit macrophage activation and activation-induced fibroblast-to-myofibroblast transdifferentiation, while reducing abnormal ECM deposition by activating the Nrf2 signaling pathway (102). Another key regulator of iron metabolism, heme oxygenase-1 (HO-1), serves as a central enzyme in heme degradation, catalyzing the conversion of heme into carbon monoxide (CO), biliverdin, and free ferrous iron ions. Downregulation of HO-1 expression in pulmonary macrophages impairs their ability to undergo M2 polarization (103–105). The use of HO-1 inhibitors can inhibit the trend toward M2 polarization (106). However, further research is needed to elucidate the specific regulatory mechanisms of HO-1 in fibrotic diseases, which may potentially serve as therapeutic targets in the future.

### 6.2 Novel therapeutic strategies and future directions

Focusing on the intersection between macrophage polarization reprogramming and iron metabolism intervention may reveal significant cross-talk between these processes. For example, miR-29a-3p promotes M2 macrophage polarization by activating the cytokine signaling suppressor (SOCS)-1/STAT6 signaling pathway (107). However, given that iron has been shown to negatively regulate miR-29a expression, iron supplementation may inhibit M2-like macrophage polarization by downregulating this microRNA (108). Research indicates that targeting the shared iron homeostasis between cells may represent a more suitable therapeutic strategy. In polystyrene nanoplastics (PS-NPs)-induced pulmonary fibrosis, treatment with the iron chelator DFO combined with the mineral absorption inhibitor esomeprazole (a proton pump inhibitor, PPI) may delay fibrosis progression by intervening in macrophage-fibroblast interactions and regulating iron homeostasis (109). However, regardless of how the target functions, delivery technology remains a challenge that must be overcome. To enhance the targeting and specificity of therapies, researchers are increasingly turning their attention to lung-directed delivery systems. These include nanoparticle delivery platforms and inhalable formulations, which offer potential solutions for improving lung tissue targeting while reducing systemic exposure risks (110). For example, iron-based nanoscale metal-organic frameworks (nMOFs) serve as immunostimulatory carriers. When conjugated with therapeutic drugs, they induce macrophages to reverse from an M2 anti-inflammatory phenotype to an M1 pro-inflammatory phenotype (111). The efficacy and safety of these innovative technologies when applied to the unique microenvironment of IPF require systematic evaluation through rigorous preclinical and clinical studies (112). Although existing preclinical evidence supports the potential of targeting iron metabolism to modulate macrophage polarization in treating IPF, translational research must proceed with caution. For example, CCR2 holds significant importance as a therapeutic target in multiple fibrotic diseases, as it not only markedly attenuates monocyte chemotaxis and aggregation but also inhibits M2 macrophage polarization (113). However, the clinical development failure of CCR2 inhibitors as treatments for liver fibrosis suggests that suppressing a single pathway alone may not yield favorable outcomes, necessitating further clinical trials for multifaceted validation (114). Similarly, iron metabolism intervention strategies may require synergistic regulation with other targets to achieve clinically meaningful therapeutic benefits. Future research may need to focus on developing more precise biomarker-guided patient stratification strategies, continuously optimizing lung-specific delivery technologies, and thoroughly exploring synergistic effects between iron metabolism regulation and existing antifibrotic drugs such as nintedanib or pirfenidone. This approach aims to provide IPF patients with more effective and safer treatment options.

## 7 Conclusion and perspectives

### 7.1 Summary of key findings

IPF is a progressive and fatal interstitial lung disease, involves complex interactions across multiple levels in its pathogenesis, including alveolar epithelial injury, immune microenvironment dysregulation, and abnormal ECM deposition. This review systematically examines the pivotal role of macrophages in IPF, particularly focusing on the critical significance of M1/M2 polarization phenotype switching and iron metabolism disorders in driving the fibrotic process. Existing research indicates that M2 macrophages directly promote fibroblast activation and ECM deposition by secreting factors such as TGF-β, PDGF, and LOXL2. Meanwhile, iron overload exacerbates M2 polarization through signaling axes like HIF-1α/STAT6. Alterations in cellular and microenvironmental iron metabolism may further intensify pulmonary interstitial fibrosis.

### 7.2 Therapeutic challenges and future perspectives

Although strategies targeting iron metabolism, such as iron chelators, antagonists of iron metabolism-related factors, and nanocarrier delivery systems, have demonstrated potential in preclinical studies, their clinical translation still faces significant challenges, including insufficient targeting, systemic toxicity, and patient heterogeneity. Future research may focus on developing patient stratification methods based on iron metabolism-related biomarkers, optimizing lung-specific delivery systems, and exploring synergistic effects between iron metabolism regulation and existing anti-fibrotic drugs. Concurrently, leveraging advanced technologies such as single-cell sequencing and spatial transcriptomics to decipher iron metabolism characteristics and microenvironment interaction mechanisms in macrophage subpopulations could provide new directions for precision therapy in IPF.Through multi-targeted, personalized, combined intervention strategies, it is anticipated that current therapeutic bottlenecks can be overcome, thereby improving the prognosis for IPF patients.
